
JOURNAL INFORMATION
==============================
NLM Title Abbreviation: J Diabetes Investig
Journal ID: J Diabetes Investig
NLM Journal ID: 101520702
Journal ID: JDI

Journal of Diabetes Investigation

ISSN: 2040-1116
EISSN: 2040-1124
Publisher: Wiley

ARTICLE INFORMATION
==============================
PMCID: PMC11474855
DOI: 10.1111/jdi.13855
Article ID: JDI13855
Article version: 1
Subjects: Issue Information, Issue Information

Issue Information

Electronic publication date: 2023 Aug 23
Publication date: 2023 Sep
Volume: 14
Issue: 9
First page: 1011
Last page: 1012
Copyright: © 2023 The Authors. Journal of Diabetes Investigation published by Asian Association for the Study of Diabetes (AASD) and John Wiley & Sons Australia, Ltd
License: This is an open access article under the terms of the http://creativecommons.org/licenses/by/4.0/ License, which permits use, distribution and reproduction in any medium, provided the original work is properly cited.
License URL: https://creativecommons.org/licenses/by/4.0/

Figure count: 0
Table count: 0
Page count: 5

source-schema-version-number: 2.0
cover-date: September 2023
details-of-publishers-convertor: Converter:WILEY_ML3GV2_TO_JATSPMC version:6.4.9 mode:remove_FC converted:15.10.2024

==============================

### JDI

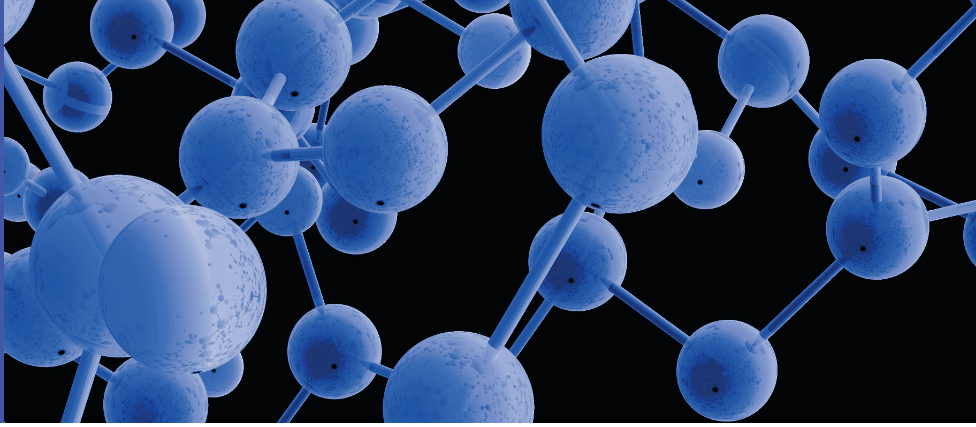

Volume 14 | Issue 9
September 2023

**Journal of Diabetes Investigation**
Official Journal of the Asian Association for the Study of Diabetes

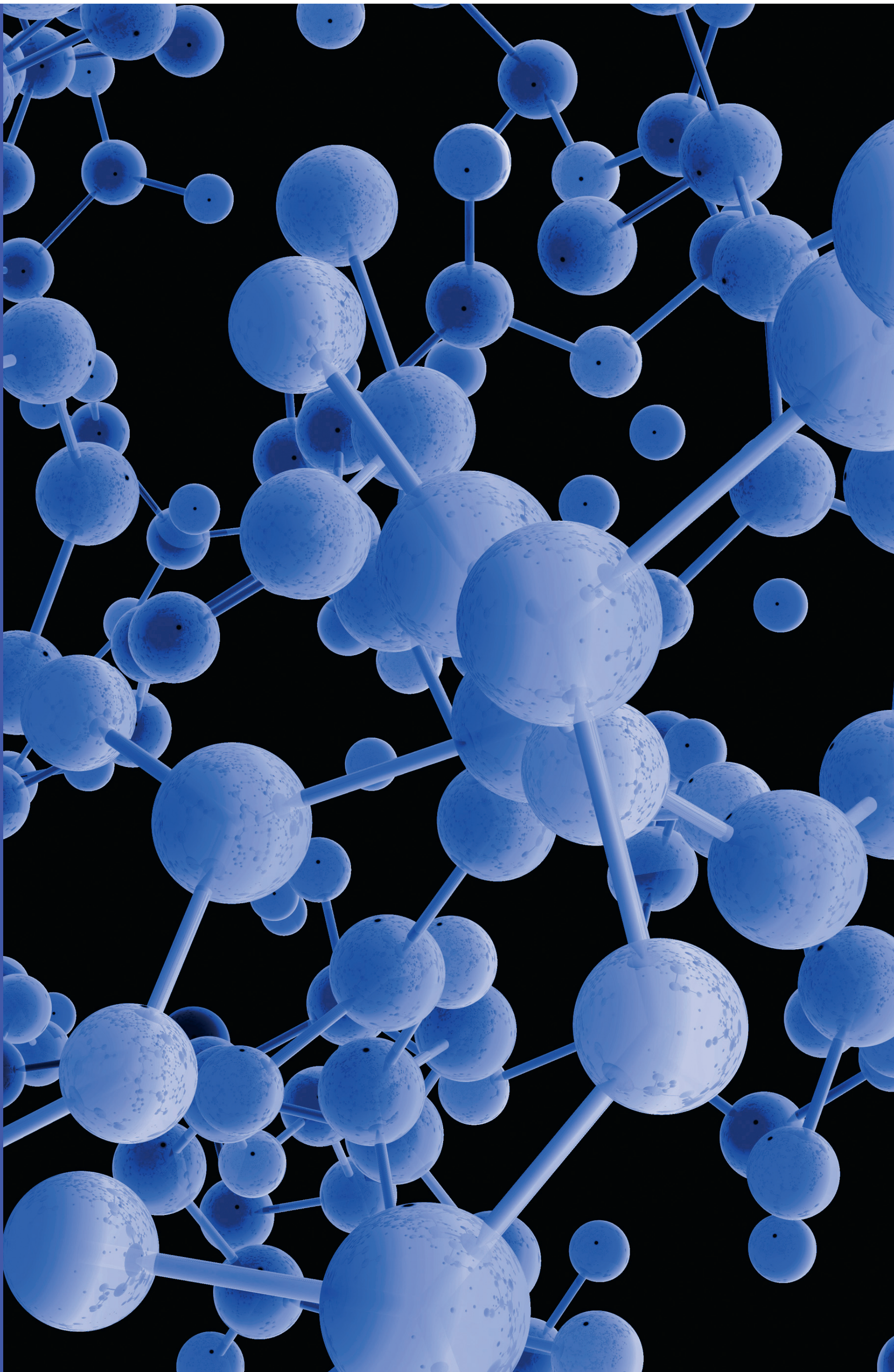

Animal names
predicting DASC-8

THE ASIAN ASSOCIATION FOR THE STUDY OF DIABETES

WILEY

### JDI Journal of Diabetes Investigation

Open access

Official Journal of the Asian Association for the Study of Diabetes

**Editor-in-Chief**
***Nigishi Hotta,*** *Japan Organization of Occupational Health and Safety, Chubu Rosai Hospital, Nagoya, Japan*

**Co-Editor**
***Domenico Accili,*** *Columbia University, New York, USA*

**Expert Editor**
***Bo Ahren,*** *Lund, Sweden*
***Young Min Cho,*** *Seoul, Korea*
***Sean Dinneen,*** *Galway, Ireland*
***Marc Gilbert,*** *Montpelier, France*
***Limei Liu,*** *Shanghai, China*
***Ronald Ma,*** *Hong Kong, China*
***Louis Philipson,*** *Chicago, USA*
***Wayne H-H Sheu,*** *Taichung, Taiwan*
***Bernard Thorens,*** *Lausanne, Switzerland*
***Daisuke Yabe,*** *Gifu, Japan*

**Editorial Board**
***Eiichi Araki,*** *Kumamoto, Japan*
***Xiaoping Chen,*** *Beijing, China*
***Lee-Ming Chuang,*** *Taipei, Taiwan*
***Masakazu Haneda,*** *Asahikawa, Japan*
***Larry Low-Tone Ho,*** *Taipei, Taiwan*
***Tianpei Hong,*** *Beijing, China*
***Akihisa Imagawa,*** *Osaka, Japan*
***Nobuya Inagaki,*** *Kyoto, Japan*
***Linong Ji,*** *Beijing, China*
***Weiping Jia,*** *Shanghai, China*
***Yi-Der Jiang,*** *Taipei, Taiwan*
***Takashi Kadowaki,*** *Tokyo, Japan*
***Kohei Kaku,*** *Kurashiki, Japan*
***Keizo Kanasaki,*** *Izumo, Japan*
***Doo-Man Kim,*** *Seoul, Korea*
***Karen Lam,*** *Hong Kong, China*
***Hong Kyu Lee,*** *Gyeonggi-do, Korea*
***In-Kyu Lee,*** *Daegu, Korea*
***Moon-Kyu Lee,*** *Seoul, Korea*
***Jiro Nakamura,*** *Nagakute, Japan*
***Kishio Nanjo,*** *Wakayama, Japan*
***Wataru Ogawa,*** *Kobe, Japan*
***Yutaka Seino,*** *Osaka, Japan*
***Shyi-Jang Shin,*** *Kaohsiung, Taiwan*
***Hirohito Sone,*** *Niigata, Japan*
***Kathryn Tan,*** *Hong Kong, China*
***Yukio Tanizawa,*** *Ube, Japan*
***Kohjiro Ueki,*** *Tokyo, Japan*
***Jun Wada,*** *Okayama, Japan*
***Hirotaka Watada,*** *Tokyo, Japan*
***Jianping Weng,*** *Guangzhou, China*
***Soroku Yagihashi,*** *Hirosaki, Japan*
***Hiroshi Yamamoto,*** *Kanazawa, Japan*
***Toshimasa Yamauchi,*** *Tokyo, Japan*
***Wenying Yang,*** *Beijing, China*
***Kazuki Yasuda,*** *Tokyo, Japan*
***Koutaro Yokote,*** *Chiba, Japan*
***Kun-Ho Yoon,*** *Seoul, Korea*
***Dalong Zhu,*** *Nanjing, China*

**International Editorial Board**
***Michael A. Brownlee,*** *New York, USA*
***Subrata Chakrabarti,*** *Ontario, Canada*
***Mark E. Cooper,*** *Prahran, Australia*
***Philippe Froguel,*** *London, UK*
***Wilfred Fujimoto,*** *Hawaii, USA*
***Michael S. German,*** *San Francisco, USA*
***Andrew Hattersley,*** *Exeter, UK*
***Jean-Claude Henquin,*** *Brussels, Belgium*
***Jens Juul Holst,*** *Copenhagen, Denmark*
***Frank B. Hu,*** *Boston, USA*
***C. Ronald Kahn,*** *Boston, USA*
***George L. King,*** *Boston, USA*
***Rayaz A. Malik,*** *Manchester, UK*
***Michael Mauer,*** *Minneapolis, USA*
***Carl Erik Mogensen,*** *Aarhus, Denmark*
***Steven E. Shoelson,*** *Boston, USA*
***Jay S. Skyler,*** *Miami, USA*
***Paul Zimmet,*** *Melbourne, Australia*

**Assistant Editorial Board**
***Tien-Jyun Chang,*** *Taipei, Taiwan*
***Yoshiyuki Hamamoto,*** *Osaka, Japan*
***Norio Harada,*** *Kyoto, Japan*
***Yi-Jen Hung,*** *Taipei, Taiwan*
***Chi-Min Hwu,*** *Taipei, Taiwan*
***Hideki Kamiya,*** *Aichi, Japan*
***Dae Jung Kim,*** *Suwon, Korea*
***Seung-Hwan Lee,*** *Seoul, Korea*
***Hung-Yuan Li,*** *Taipei, Taiwan*
***Takashi Matsuzaka,*** *Tsukuba, Japan*
***Keiko Naruse,*** *Aichi, Japan*
***Takeshi Nishikawa,*** *Kumamoto, Japan*
***Eung-Jung Rhee,*** *Seoul, Korea*
***Yusuke Seino,*** *Aichi, Japan*
***Hironori Waki,*** *Tokyo, Japan*

**Aims and Scope:** *Journal of Diabetes Investigation* is your core diabetes journal from Asia; the official journal of the Asian Association for the Study of Diabetes (AASD). The journal publishes original research, country reports, commentaries, reviews, mini-reviews, case reports, letters, as well as editorials and news. The journal embraces clinical and experimental research in diabetes and related areas. This includes aspects of prevention, treatment, as well as molecular aspects and pathophysiology. Translational research focused on the exchange of ideas between clinicians and researchers is also welcome.

**Address for Editorial Correspondence:**
The Editor, *Journal of Diabetes Investigation*, Nomura Fudosan Nishi Shinjuku Building, 8F 8-4-2 Nishi Shinjuku, Shinjuku-ku, Tokyo 160-0023, Japan. Tel: +81 3 4520 9009; Fax: +81 3 4520-9059 Email: JDI@wiley.com

**Copyright © 2023 Asian Association for the Study of Diabetes and John Wiley & Sons Australia, Ltd**

**Cover:** Insulin molecule © Pasieka/Science Photo Library

This journal is available online at Wiley Online Library. Visit wileyonlinelibrary.com to search the articles and register for table of contents and e-mail alerts.

**For submission instructions, and all other information visit wileyonlinelibrary.com/journal/jdi**

ISSN 2040-1124 (Online)

JDI.JEB.Sep23

WILEY

**Publisher**
*Journal of Diabetes Investigation* is published by John Wiley & Sons Australia, Ltd
Level 19, 15 William Street,
Melbourne, Victoria 3000,
Australia.
Tel: +61 (0)3 9274 3100

**Production Editor**
Jayalakshmi Mohan (email: JDIproduction@wiley.com)

**Abstracting and Indexing Services**
The Journal is indexed by Academic OneFile (GALE Cengage), Biological Abstracts (Clarivate Analytics), Biological Science Database (ProQuest), BIOSIS Previews (Clarivate Analytics), Embase (Elsevier), Health & Medical Collection (ProQuest), Health Research Premium Collection (ProQuest), Hospital Premium Collection (ProQuest), MEDLINE/PubMed (NLM), Natural Science Collection (ProQuest), ProQuest Central (ProQuest), Science Citation Index Expanded (Clarivate Analytics), SciTech Premium Collection (ProQuest), SCOPUS (Elsevier), Web of Science (Clarivate Analytics).

**Open Access and Copyright**
All articles published by *Journal of Diabetes Investigation* are fully open access: immediately freely available to read, download and share. All *Journal of Diabetes Investigation* articles accepted from 24 September 2013 are published under the terms of the Creative Commons Attribution 4.0 Unported (CC BY 4.0) license, which permits use, distribution and reproduction in any medium, provided the original work is properly cited. Articles accepted before this date were published under the agreement as stated in the final article. Copyright on any *Journal of Diabetes Investigation* article accepted after 24 September 2013, the date on which *Journal of Diabetes Investigation* became an open access journal, is retained by the author(s). Authors grant to *Journal of Diabetes Investigation* and Wiley a license to publish the article and for Wiley to identify itself as the original publisher. Authors also grant any third party the right to use the article freely as long as its integrity is maintained and its original authors, citation details and publisher are identified. Please visit https://authorservices.wiley.com/author-resources/Journal-Authors/open-access/hybrid-open-access.html for further information about Open Access.

**Use by commercial ''for-profit'' organisations (not applicable to CC BY 4.0 licensed articles)**
Use of Wiley Open Access articles for commercial, promotional, or marketing purposes requires further explicit permission from Wiley and will be subject to a fee. Requests to reproduce material from this journal content are being handled through the Rightslink® online service.

- Locate the article you wish to reproduce on Wiley Online Library (http://onlinelibrary.wiley.com)
- Navigate to the abstract page
- Click on the 'Request Permissions' link, under the 'ARTICLE TOOLS' menu
- Follow the online instructions and select your requirements from the drop down options and click on 'quick price' to get a quote
- Create a RightsLink® account to complete your transaction (and pay, where applicable)
- Read and accept our Terms & Conditions and Download your license
- For any technical queries please contact customercare@copyright.com
- For further information and to view a Rightslink® demo please visit www.wiley.com and select Rights & Permissions.
- Print reprints of Wiley Open Access articles can be purchased from corporatesales@wiley.com.

**Disclaimer**
The Publisher, the Asian Association for the Study of Diabetes, and Editors cannot be held responsible for errors or any consequences arising from the use of information contained in this journal; the views and opinions expressed do not necessarily reflect those of the Publisher, the Asian Association for the Study of Diabetes, or Editors, neither does the publication of advertisements constitute any endorsement by the Publisher, the Asian Association for the Study of Diabetes, or Editors of the products advertised.

Wiley Open Access articles posted to repositories or websites are without warranty from Wiley of any kind, either express or implied, including, but not limited to, warranties of merchantability, fitness for a particular purpose, or noninfringement.
To the fullest extent permitted by law Wiley disclaims all liability for any loss or damage arising out of, or in connection, with the use of or inability to use the content.

**Journal Customer Services:** For ordering information, claims and any enquiry concerning your journal subscription please go to https://wolsupport.wiley.com/s/contactsupport?tabset-a7d10=2 or contact your nearest office.
**Americas:** Email: cs-journals@wiley.com; Tel: +1 877 762 2974
**Europe, Middle East and Africa:** Email: cs-journals@wiley.com; Tel: +44 (0) 1865 778315; 0800 1800 536 (Germany)
**Germany, Austria, Switzerland, Luxembourg, Liechtenstein:** cs-germany@wiley.com; Tel: 0800 1800 536 (Germany)
**Asia Pacific:** Email: cs-journals@wiley.com; Tel: +65 3165 0890.
**Japan:** For Japanese speaking support, Email: cs-japan@wiley.com; .
**Visit our Online Customer Help** at https://wolsupport.wiley.com/s/contactsupport?tabset-a7d10=2

Wiley's Corporate Citizenship initiative seeks to address the environmental, social, economic, and ethical challenges faced in our business and which are important to our diverse stakeholder groups. Since launching the initiative, we have focused on sharing our content with those in need, enhancing community philanthropy, reducing our carbon impact, creating global guidelines and best practices for paper use, establishing a vendor code of ethics, and engaging our colleagues and other stakeholders in our efforts. Follow our progress at www.wiley.com/go/citizenship

VOLUME 14 – ISSUE 9 – SEPTEMBER 2023

### Journal of Diabetes Investigation

Open access

### Review

**Contribution of animal models to diabetes research: Its history, significance, and translation to humans**
Soroku Yagihashi 1015

### Commentary

**Impact of the angiotensin receptor-neprilysin inhibitor in clinical diabetes management: Potential benefits and pitfalls**
Tomoko Kato, Takaaki Murakami, Daisuke Yabe, and Norio Harada 1038

### Update

**Updates on dyslipidemia in patients with diabetes**
Shintaro Ide, Yoshiro Maezawa, and Koutaro Yokote 1041

### Original Articles

### Basic Science and Research

**Blockade of glucagon increases muscle mass and alters fiber type composition in mice deficient in proglucagon-derived peptides**
Shinji Ueno, Yusuke Seino, Shihomi Hidaka, Masashi Nakatani, Keisuke Hitachi, Naoya Murao, Yasuhiro Maeda, Haruki Fujisawa, Megumi Shibata, Takeshi Takayanagi, Katsumi Iizuka, Daisuke Yabe, Yoshihisa Sugimura, Kunihiro Tsuchida, Yoshitaka Hayashi, and Atsushi Suzuki 1045

**LncRNA SNHG1 knockdown inhibits hyperglycemia induced ferroptosis *via* miR-16-5p/ACSL4 axis to alleviate diabetic nephropathy**
Xiangdong Fang, Jianling Song, Yanxia Chen, Shuying Zhu, Weiping Tu, Ben Ke, and Lidong Wu 1056

**miR-31 ameliorates type 2 diabetic vascular damage through up-regulation of hypoxia-inducible factor-1α/vascular endothelial growth factor-A**
Yuan Fu, Ruochen Du, Yufei Wang, Yitong Yuan, Yujuan Zhang, Chunfang Wang, and Xuanping Zhang 1070

### Clinical Science and Care

**Improving diagnostic accuracy of 3 Screen ICA ELISA kit in the identification of Japanese type 1 diabetes**
Eiji Kawasaki, Hideaki Jinnouchi, Yasutaka Maeda, Akira Okada, Yoshihisa Ito, and Koichi Kawai 1081

**A surge in serum mucosal cytokines associated with seroconversion in children at risk for type 1 diabetes**
Leonard C Harrison, Esther Bandala-Sanchez, Helena Oakey, Peter G Colman, Kelly Watson, Ki Wook Kim, Roy Wu, Emma E Hamilton-Williams, Natalie L Stone, Aveni Haynes, Rebecca L Thomson, Peter J Vuillermin, Georgia Soldatos, William D Rawlinson, Kelly J McGorm, Grant Morahan, Simon C Barry, Richard O Sinnott, John M Wentworth, Jennifer J Couper, Megan AS Penno, on behalf of the ENDIA Study Group 1092

**Effect of patient characteristics on the efficacy and safety of imeglimin monotherapy in Japanese patients with type 2 diabetes mellitus: A post-hoc analysis of two randomized, placebo-controlled trials**
Katsuhiko Hagi, Kenji Kochi, Hirotaka Watada, Kohei Kaku, and Kohjiro Ueki 1101

**Ultra-high performance liquid chromatography coupled to tandem mass spectrometry-based metabolomics study of diabetic distal symmetric polyneuropathy**
Jiahui Xu, Qingguang Chen, Mengjie Cai, Xu Han, and Hao Lu 1110

### Clinical Trial

**Predicting three categories of Dementia Assessment Sheet for Community-based Integrated Care System 8-items score-based glycemic targets using the number of animal names recalled**
Hiroko Hiiragi, Taichi Minami, and Yasuo Terauchi 1121

### Original Article

**Validity of the short-form five-item Problem Area in Diabetes questionnaire as a depression screening tool in type 2 diabetes mellitus patients**
Donovan Tay, Marvin Chua, and Joan Khoo **1128**

### Case Report

**Weight loss improves inflammation by T helper 17 cells in an obese patient with psoriasis at high risk for cardiovascular events**
Yoshiro Maezawa, Yusuke Endo, Satomi Kono, Tomohiro Ohno, Yuumi Nakamura, Naoya Teramoto, Ayano Yamaguchi, Kazuto Aono, Takuya Minamizuka, Hisaya Kato, Takahiro Ishikawa, Masaya Koshizaka, Minoru Takemoto, Toshinori Nakayama, and Koutaro Yokote **1136**
